# Robotic Flexible Ureteroscopy: Systematic Review and Meta-Analysis of Surgical Efficacy, Safety and Ergonomic Outcomes

**DOI:** 10.7759/cureus.90447

**Published:** 2025-08-18

**Authors:** Praveen Gopi, Muhammed Ishfaq, Zakaria W Shkoukani, Ninaad Awsare, John McCabe, Azi Samsudin, Kaylie E Hughes, Mohamed Abdulmajed

**Affiliations:** 1 Department of Urology, Mersey and West Lancashire Teaching Hospitals National Health Service (NHS) Trust, Prescot, GBR

**Keywords:** ergonomics, meta-analysis, renal calculi, robotic ureteroscopy, stone-free rate

## Abstract

Robotic flexible ureteroscopy (RFURS) has emerged as a novel approach to address the ergonomic challenges and technical limitations of conventional flexible ureteroscopy (FURS) for renal stone management. While FURS remains a cornerstone in treating nephrolithiasis, prolonged procedures contribute to surgeon fatigue, musculoskeletal strain, and increased radiation exposure. Despite growing adoption, the literature lacks a synthesis of the clinical benefits, cost-effectiveness, and long-term outcomes of RFURS compared to conventional approaches.

The objective of our study is to synthesize the existing evidence in the literature and produce a comprehensive systematic review of RFURS.

Following the Preferred Reporting Items for Systematic Reviews and Meta-Analyses (PRISMA) guidelines, we searched PubMed, Embase, and Cochrane (inception: June 2025) for clinical studies on RFURS. Meta-analysis used random-effects models for pooled estimates of stone-free rates (SFRs), operative times and complications. The risk of bias was assessed by the Newcastle-Ottawa Scale and the Cochrane risk tool.

Twelve studies (706 patients) were included. RFURS achieved a pooled SFR of 87.4% (95% confidence interval (CI): 82.7-92.0%), comparable to conventional FURS. Pooled operative time was 94.7 minutes (95% CI: 78.9-110.5), longer than conventional FURS. Complication rates were 10.6% (95% CI: 5.1-16.1%) similar to conventional FURS. Ergonomics were superior, with reduced surgeon fatigue and radiation exposure. Learning curves vary according to the robot platforms and early proficiency is noted among experienced endoscopists. Cost-effectiveness data were limited.

RFURS demonstrates non-inferior efficacy and safety to conventional FURS, with enhanced ergonomics and manageable learning curves. High heterogeneity and limited cost data necessitate larger comparative studies.

## Introduction and background

The management of renal stones has evolved significantly over recent decades, with flexible ureteroscopy (FURS) becoming the mainstay due to its minimally invasive nature and high success rates. However, FURS is associated with several challenges, including surgeon fatigue, limited instrument maneuverability, radiation exposure during prolonged procedures and specific ergonomics issues such as awkward hand and wrist positions, repetitive movements, non-neutral body postures (including extended standing or uncomfortable seating) and substantial musculoskeletal strain to the neck, back, shoulder and upper extremities [1]. To address these limitations, robotic flexible ureteroscopy (RFURS) has been introduced, offering enhanced ergonomics, improved precision, and potentially greater procedural efficiency [2]. Various robotic platforms currently available provide surgeons with better control and stability, which may translate into improved patient outcomes [1]. Early studies report comparable stone-free rates (SFRs) between RFURS and FURS (70%-92.4%) and highlight novel metrics like stone treatment efficiency (STE) and composite endpoints such as the tetrafecta (complete clearance, no complications, no auxiliary procedures, same-day discharge) [1]. Despite promising early results, the clinical benefits, safety profile and cost-effectiveness of RFURS compared to traditional FURS remain to be fully clarified [2]. This article aims to provide a comprehensive overview of RFURS technology, evaluate current evidence on its efficacy and safety in treating renal stones and discuss future directions for research and clinical practice.

Current studies on RFURS are limited by small sample sizes, single-arm designs and variability in outcome measures, which hinder robust comparisons with FURS [3]. For instance, while the Avicenna Roboflex™ ((ELMED, Ankara, Türkiye)) demonstrates promising ergonomic advantages, its cost-effectiveness and learning curve remain understudied [4]. Additionally, recent proposals for standardised composite endpoints (e.g., tetrafecta) are not universally adopted, complicating cross-study comparisons [1]. A meta-analysis is critical to consolidate fragmented evidence, quantify the efficacy and safety of RFURS and identify gaps in understanding its role within modern endourology.

The objective of this study is to provide a comprehensive assessment of both clinical efficacy (including SFRs, operative times and complications) and ergonomic impact, learning curve and cost efficiency of RFURS in order to inform best practices and guide future adoption of robotic technologies in endourology.

## Review

Methods

Protocol and Registration

This systematic review and meta-analysis was conducted following the Preferred Reporting Items for Systematic Reviews and Meta-Analyses (PRISMA) guidelines. The protocol was prospectively registered with the International Prospective Register of Systematic Reviews (PROSPERO) (CRD420251077721).

Eligibility Criteria

Table 1 describes the eligibility criteria used in this study.

**Table 1:** Eligibility criteria

Criteria Type	Description
Inclusion Criteria	1. Original clinical data on robotic retrograde intrarenal surgery (robotic flexible ureteroscopy (RFURS)), including endoscopic intrarenal surgery (ECIRS), for the management of renal stones in human subjects. 2. Studies with or without a comparator group, such as conventional flexible ureteroscopy (FURS). 3. Studies with provided quantitative and qualitative outcomes, including stone-free rate (SFR), operative time, complication rates, total hospital stay, ergonomics assessment, cost-effectiveness, learning curve for RFURS. 4. Articles written in English. 5. Published in peer-reviewed journals. 6. Study Designs: Randomised controlled trials (RCTs), cohort studies, case-control studies and case series.
Exclusion Criteria	1. Studies involving animal subjects 2. Papers purely technical without reporting clinical outcomes. 3. Reviews, editorials, conference abstracts and case reports. 4. Studies lacking extractable outcome data relevant to the review objectives.

Literature Search Strategy

A comprehensive literature search was conducted in Medline (via PubMed), Embase and the Cochrane Library from inception to June 1, 2025. The search strategy combined terms related to “robotic flexible ureteroscopy,” “RFURS,” “renal stones,” and specific robotic platforms (e.g., “Avicenna Roboflex,” ILY”). Reference lists of included articles and relevant reviews were hand-searched for additional studies. The full search strategy is provided in Supplementary Material 1 (see Appendices).

Study Selection

All identified records were imported into a reference management software and duplicates were removed. Two independent reviewers (P.G. and M.I.) screened titles and abstracts for eligibility. Full texts of potentially relevant studies were assessed for inclusion. Discrepancies were resolved by consensus or consultation with a third reviewer (Z.S.).

Data Extraction Process

Data were independently extracted by two reviewers (P.G. and M.I.) using a standardised data collection form in Microsoft Excel (Microsoft, Redmond, NY, USa) to ensure consistency and minimise bias.

(a) *Study characteristics: *The extracted variables included study characteristics such as the name of the author, year of publication, country of origin, study design and sample size. 

(b) *Patient demographics: *Patient-related data were collected on age, sex and stone characteristics.

(c) *Intervention details: *Information on the specific robotic system used, the presence of a comparator (if applicable), ureteric access sheath usage and pre-stenting status were recorded for each study.

(d) *Primary outcomes: *Key clinical outcomes included SFRs, operative time, complication rates (both overall and stratified according to the Clavien-Dindo classification), hospital stay duration, follow-up period, and the type of follow-up imaging used. Where applicable, the presence of clinically significant residual fragments (CIRF) was also documented.

(e) *Secondary outcomes: *Secondary measures encompassed ergonomic assessments, cost-related data, and reported learning curve metrics.

Risk of Bias Assessment

The methodological quality and risk of bias were evaluated using the Newcastle-Ottawa scale for all observational studies [4]. This uses a star scoring system with a maximum total score of 9 for each study. The score is calculated by awarding stars based on specific criteria within the three domains, with up to four stars for selection, two for comparability, and three for exposure. In each domain, there are clear criteria for each star. A study with more stars is considered to have met more of the criteria for good methodological quality. The Cochrane risk of bias tool was reserved for the assessment of randomised trials [5].

Data Synthesis and Statistical Analysis

Meta-analysis was performed for primary outcomes reported in three or more studies. Continuous data (operative time) were synthesised using mean values, while dichotomous data (SFR, complications), in single-arm studies, were used as untransformed proportions (PR). All analyses were conducted in Open MetaAnalyst software (Center for Evidence-Based Medicine, Brown University, Providence, RI, USA), with results visualised in forest plots (95% confidence intervals (CIs)).

Heterogeneity Assessment

Heterogeneity was quantified via the Cochran Q test (χ²) and I² statistic, interpreted as: 0%-25%: low heterogeneity, 25%-75%: moderate heterogeneity, >75%: substantial heterogeneity. To address substantial heterogeneity, a random-effects model or inverse variance method was applied, accounting for inter-study variance in the pooled effect estimates. The median values were converted to mean values for statistical analysis using Hozo et al. [6] and Wan et al. [7] methods.

Certainty of Evidence

The certainty of evidence was assessed using the Grading of Recommendations Assessment, Development and Evaluation (GRADE) approach [8] and results are provided in Supplementary Table 6.

Ethics Statement

As this review utilised published data, ethical approval was not required.

Results

Study Selection

**Figure 1:**
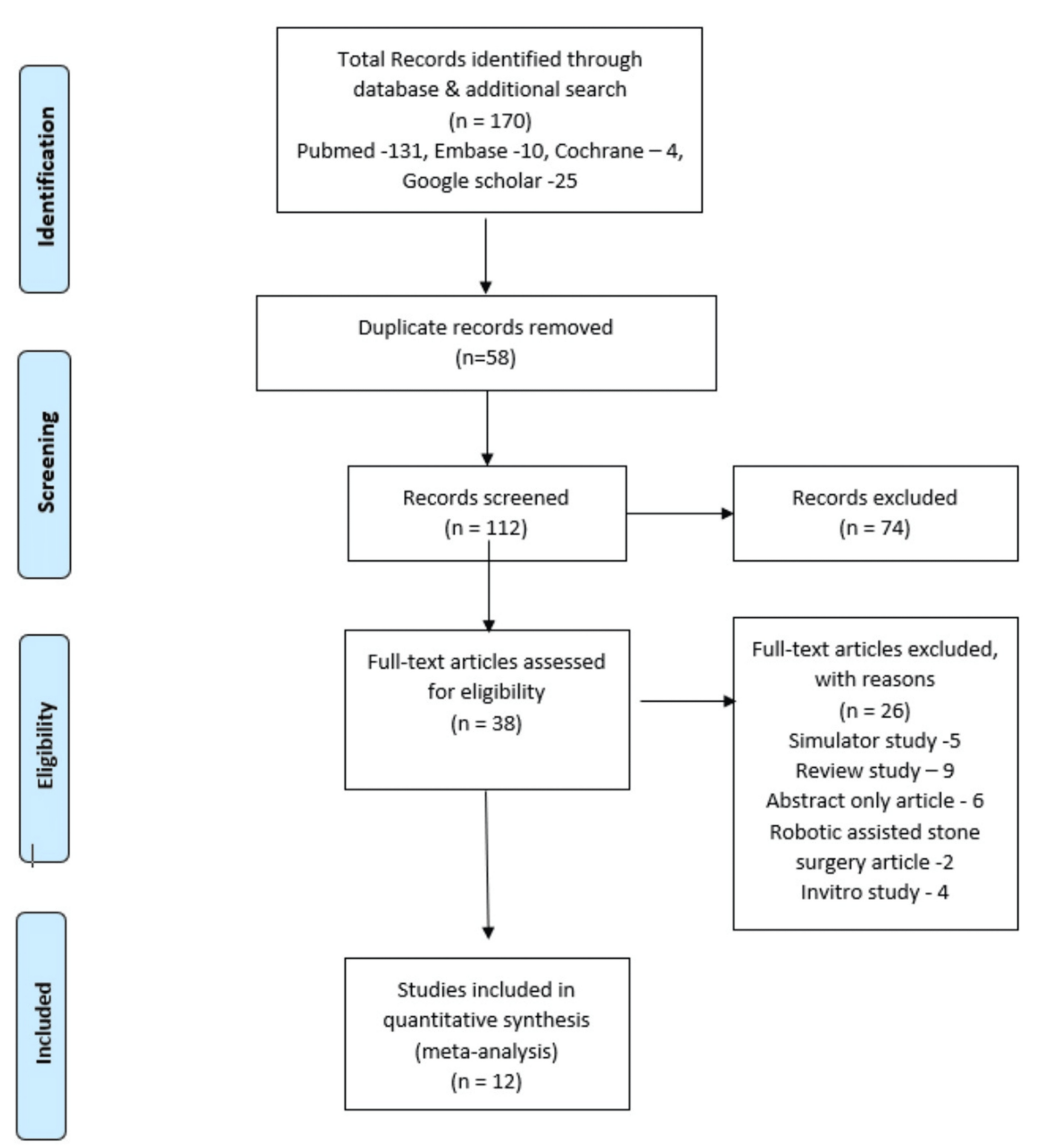
PRISMA flow chart for article selection and screening in assessing the surgical efficacy, safety and ergonomic outcomes. PRISMA: Preferred Reporting Items for Systematic Reviews and Meta-Analyses.

The PRISMA flow diagram (Figure 1) systematically outlines the study selection process for this review, adhering to PRISMA 2020 guidelines. Initial searches were conducted by independent authors (P.G. and M.I.) across Medline, Embase and Cochrane Central and yielded 170 records. After duplicate removal (58 excluded), 112 unique records underwent title/abstract screening, with 74 excluded for irrelevance (e.g., non-robotic interventions, animal studies). The remaining 38 full-text articles were assessed for eligibility, and 26 were excluded due to: in vitro study (n=4), robotic-assisted stone surgery (n=2), simulator study (n=5), review study (n=9), abstract-only data (n=6). Twelve studies (comprising clinical studies) met the inclusion criteria for quantitative synthesis and were included in the meta-analysis. Figure 1 transparently maps attrition at each phase, with exclusion reasons explicitly documented to minimise selection bias.

**Table 2:** Summary of the basic demographics of all studies included in this systematic review and meta-analysis. The 12 studies included nine observational, two clinical trial and one comparative study with conventional flexible ureteroscopy. * Median value. NR: Not reported. mECIRS: Mini-endoscopic combined intrarenal surgery (FURS+PCNL); FURS: flexible ureteroscopy; RFUS: robotic flexible ureteroscopy; PCNL: percutaneous nephrolithotomy; UAS: ureteric access sheath; SD: standard deviation.

Author	Year	Journal	Country	(N) Patients/(N) Procedures	Age (yrs)	Study Design	Robotic machine	UAS	(N) Pre-Stenting/(N) patients
Desai et al. [9]	2011	Journal of Urology	USA	18/18	46 (26-74)	Prospective pilot feasibility study	Sensei	Flexible catheter system - 14/12 fr	18/18
Saglam et al. [10]	2014	European Urology	Turkey	81/81	42 (6-68) SD:25.4	Prospective, multicentre, observational study	Avicenna Roboflex	Yes (size NR)	NR
Geavlete et al. [11]	2016	Chirurgia	Romania	66 (RFURS)	51 (25-74)	Prospective randomised controlled trial	Avicenna Roboflex	NR	66/66
				66 (FURS)	48 (26-77)		Storz XC		
Klein et al. [12]	2021	Journal of Robotic Surgery	Germany	240/240	55.7±17.24 (18-87)	Prospective cohort study	Avicenna Roboflex	12/14Ch	225/240
Tokatli et al. [13]	2022	Journal of Lapendoscopic and Advanced Surgery Techniques	Turkey	42/44 -Robot-assisted mECIRS	42.3±12.8	Retrospective cohort study	Avicenna Roboflex	10.7/12.7	NR
Firori et al. [14]	2023	Minerva Urology and Nephrology	Italy	3/4	57	Prospective case series	ILY	10/12fr	NR
Laszkiewicz et al. [15]	2024	Central European Journal of Urology	Poland	57/46 RFURS, 11 Robot-assisted mECIRS	46 (18-82) SD: 19.3	Prospective case series	ILY	10.7/12.7 Fr	37/46
Salah et al. [1]	2024	Journal of Robotic Surgery	Qatar	100/100	40.7±9.2	Retrospective cohort study	Avicenna Roboflex	Yes (size NR)	58/100
El- Hajj et al. [16]	2024	World Journal of Urology	Lebanon	29/31	56 (44.5- 64.5)*	Prospective single-centre clinical study	ILY	Yes (size NR)	3/29
Farre et al. [17]	2024	British Journal of Urology International	Spain	6/6	62 (50-72)	Prospective case series	ILY	10/12 Fr	NR
Landman et al. [18]	2024	Journal of Urology	USA	13/13 - ECIRS	65 (35-72)*	Prospective, first-in-human clinical trial (single-arm)	Monarch	12/14 Fr	NR
Kim et al. [19]	2025	Scientific Reports	South Korea	46/46	57.5 (48.25- 63)*	Prospective, multi-centre, single-arm clinical trial	Zamenix R	11/13 Fr	46/46

Study Characteristics

A total of 12 studies encompassing 701 patients were included, traversing publication years from 2011 to 2025 and representing diverse geographic regions, including Turkey, the United States, several European countries (Poland, Italy, Spain, Romania), Lebanon, Qatar and South Korea. Study sample sizes ranged widely, from small pilot investigations to large multicentre cohorts (four to 240 patients per study). The Avicenna Roboflex platform was utilised in five studies, while four studies employed the ILY® system (Sterlab, Vallauris, France); the Zamenix R (Roen Surgical Inc., Daejeon, Korea), Sensei (Sensei Robotics, San Fransico, CA, USA) and Monarch™ platform (Ethicon/Auris Health, Raritan, NJ, USA) robots were each featured in one study. The mean age of participants ranged from 40.7 to 62.7 years. The reported mean stone sizes varied between 11.7 and 35.6 mm, with stone volumes ranging from 349 to 1,798 mm³. The use of a ureteric access sheath (UAS) was documented in 10 studies. Among studies reporting pre-stenting, 453 out of 545 patients (83.1%) underwent stent placement prior to robotic ureteroscopy.

Primary Outcomes

**Figure 2:**
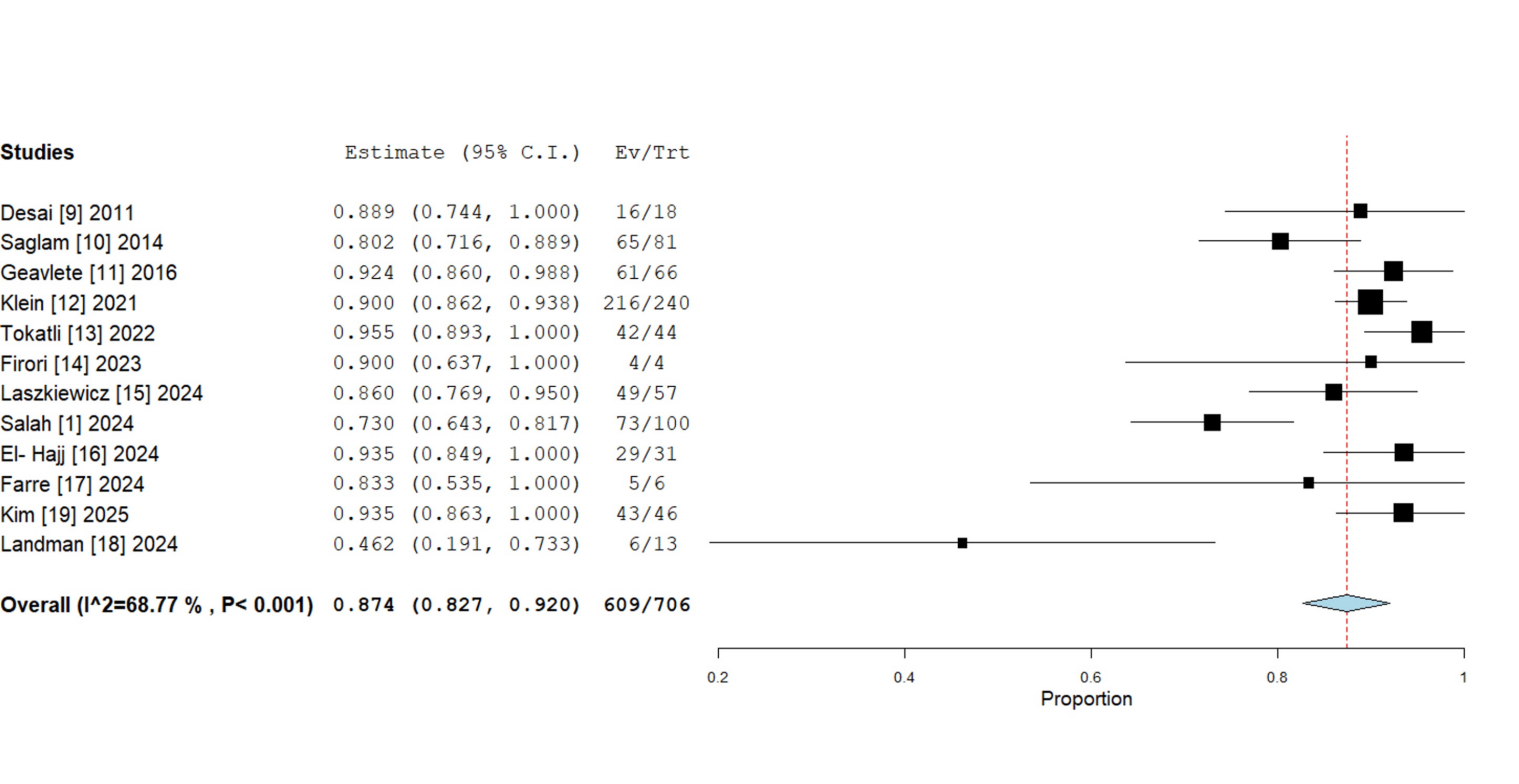
Forest plot of stone-free rate (SFR) in robotic flexible ureteroscopy, proportions with 95% confidence intervals (random-effects meta-analysis). The pooled SFR result is 87.4%, which is statistically significant (P<0.001).

*Stone-free rates (SFRs): *Across 12 included studies (706 procedures), the pooled SFR of 87.4% (95% CI: 82.7%-92.0%) was achieved, indicating high procedural efficacy. Individual study SFRs ranged from 73.0% to 95.5%, with moderate heterogeneity observed (I²=68.77%, P<0.001) (Figure 2). Notably, nine of 12 studies reported SFRs exceeding 85%, demonstrating consistent success across diverse clinical settings. Follow-up imaging protocols were variable, with computed tomography (CT) serving as the most common imaging modality for outcome assessment, with ultrasound (USG), intravenous pyelography (IVP) and plain radiography used as alternatives. Follow-up intervals spanned two weeks to three months postoperatively. Significant variation existed in defining clinically insignificant residual fragments (CIRFs), with thresholds ranging from 2 to 4 mm across studies (Table 3).

**Table 3:** Perioperative and clinical outcomes of robotic flexible ureteroscopy NR=Not reported. *Median value. IVP: Intravenous pyelogram; USG: ultrasound sonography; CT: computed tomography; CD: Clavien Dindo classification; CIRF: clinically insignificant residual fragments; ESWL: extracorporeal shockwave lithotripsy.

Author	Year	Operative Time (minutes)	Docking Time ( minutes)	Stone Volume (mm³)	Stone Size (mm)	Stone Free Rate	Follow-up imaging/CIRF (mm)	Hospital Stay	Follow Up	Complication
Desai et al. [9]	2011	91.3 (60-130)	7.3 min (4-18)		11.8 (9-25)	89%	IVP/NR	2-7 days	3 months	2 - Urinary tract infection (UTI); 1 - transient upper limb paresis in a kyphoscoliosis patient
Saglam et al. [10]	2014	74 ±31.8 (74-182)	59.6 s (35- 124)	1296 (432-3100) SD: 544.3	13±5.3 (5-30)	80%	X-Ray and USG/3 mm	NR	3 months	NR
Geavlete et al. [11]	2016	51 (38-103)	NR	NR	24 (10-37)	92.4%	NR/3 mm	NR	3 months	0%
		50 (41-115)			21 (11-36)	89.4%				1.5%
Klein et al. [12]	2021	91±49.95 (10-269)	5 min (1-29)	1798 mm³	NR	90%	US/KUB, 2 mm	1.5 days (1-15)	3 months	CDII -15 (Bleeding, stent symptoms, hydronephrosis) CDII - 10 (bleeding with transfusion, UTI, fever) CDIV – 1 (UTI with sepsis) CD V -2 (MI, urosepsis)
Tokatli et al. [13]	2022	103.7±20.6		NR	28.4±4.6	95.5%	NCCT/any size	43±5.8 hrs	NR	CD I – 3 (fever, hematuria)
Firori et al. [14]	2023	70.75±17.58	3 min	NR	13	100%	USG/NR	1 day	1 month	NIL
Laszkiewicz et al. [15]	2024	63(15- 91) - Robo URS 55 (32-83) - mECIRS	73 s (32- 124)		13 (0.8-2.3) SD: 0.41 19 (1.1-5.6) SD: 1.33	80.4% - RoboURS 90.9% - mECIRS	Endoscopically and Fluroscopic/4 mm	NR	NR	NR
Salah et al. [1]	2024	116 (97- 148)*	7.8±3.2 min	916 (421- 12,235)	11.7±5.8	73%	Non-contrast CT/2 mm	9.3 hrs (5.4- 166)	1 month	CD1 – 5 ( Fever, Hematuria, Stent symptoms) CDII -2 ( UTI) CDIII – 1 (Ureteric injury)
El- Hajj et al. [16]	2024	85 (60.5-100)*	3.5 min (3-5)*	736 mm³ (435-1696)*	13 (12-20)*	93.55%	X-Ray or CT/any size	1 day	2-3 Weeks	CDI – 9.68% (n=3) Pain
Farre et al. [17]	2024	77.5 (65-90)*	NR	NR	13.5 (11-15)*	83.3%	CT/2 mm	1 day (1-4)	3 months	CDII – fever (n=1) CDIIIa- psudoaneurysm after infundibulotomy requiring selective embolisation.
Landman et al. [18]	2024	183 (83-383)*	9 min (4-19)	1645.9 (523.9- 8095.6)	32.8 (11.8 -65.2)*	Grade A – 38.5% Grade B – 46.1% Grade C – 61.5%	CT/ Grade A - No CIRF; Grade B - <2 mm; Grade C - <4 mm	1 day (1-2)	30 days	UTI - 2, Ureteric injury, Grade 2
Kim et al. [19]	2025	91.5 (64.25-113.75)*	NR	349 mm³ (201- 704)*	13.7 (10-16)*	93.4%	CT/4 mm	NR	3 months	CD II - 17.39%, ureteric injury (n=8) due to UAS insertion, CD II - UTI (6.52%, n=3)

**Figure 3:**
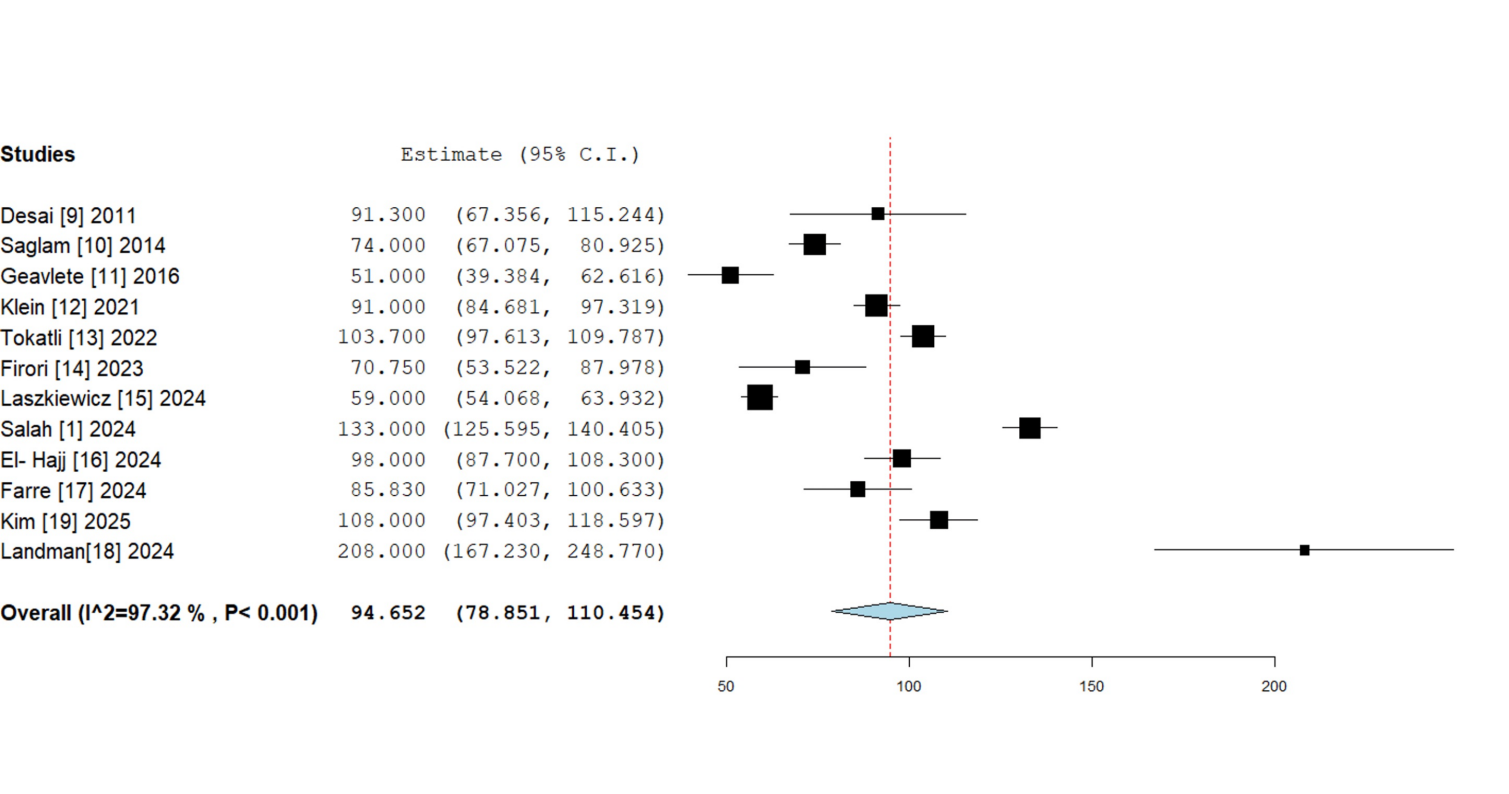
Forest plot of operative time in robotic flexible ureteroscopy, mean with 95% confidence intervals (random-effects meta-analysis). The pooled mean operative time is 94.7, which is statistically significant (P<0.001).

*Operative times: *An analysis of operative times across 12 studies revealed a pooled mean of 94.7 minutes (95% CI: 78.9-110.5 minutes). Substantial heterogeneity was observed (I²=97.32%, P<0.001), with individual study means ranging from 51.0 to 208.0 minutes (Figure 3). The shortest operative time (51 minutes) was reported by Geavlete et al. [11], while the longest (208 minutes) was documented by Landman et al. [18]. This variability likely reflects differences in robotic platforms, stone complexity, surgeon experience and procedural definitions.

**Figure 4:**
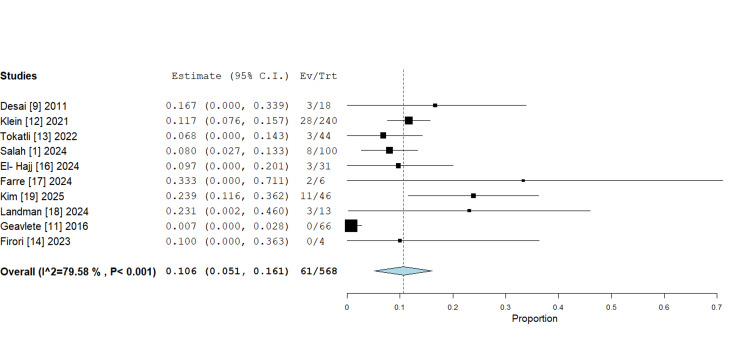
Forest plot of complication rates in robotic flexible ureteroscopy, proportion with 95% confidence intervals (random-effects meta-analysis). The pooled complication rate is 10.6%, which is statistically significant (P<0.001).

*Complication rates: *A pooled analysis of 10 studies encompassing 568 patients yielded an overall complication rate of 10.6% (95% CI: 5.1%-16.1%). Complication rates in individual studies varied widely, ranging from 0.7% to 33.3%, with significant heterogeneity detected (I²=79.58%, P<0.001) (Figure 4). Notably, two studies (Geavlete et al. [11] and Firori et al. [14]) reported no complications, whereas the highest rate was observed in the study by Farré et al. [17], at 33.3%, though this was based on a small sample size.

Secondary Outcomes

*Ergonomics: *Robotic systems consistently demonstrate superior ergonomics compared to FURS. Surgeons report reduced physical fatigue, improved posture, and less musculoskeletal strain, especially during prolonged or complex procedures. Ergonomics were specifically addressed in eight of the 12 included studies. Of these, five studies incorporated objective assessments using tools such as the visual analogue scale, surgeon satisfaction questionnaires and Likert scale surveys. All objective measures indicated a favourable ergonomic profile for robotic instrumentation, underscoring its positive impact on surgeon comfort and procedural sustainability (Table 4).

**Table 4:** Comparative assessment of ergonomics, instrumentation, cost efficiency and learning curve in robotic flexible ureteroscopy. FURS: Flexible ureteroscopy. The studies Desai et al. [9], Saglam et al. [10], Firori et al. [14], Farré et al. [17], Kim et al. [19] used various scoring systems for quantitative assessment of the ergonomics: measuring pain, fatigue, numbness etc. The Likert-type scale in Farré et al. [17] measured manageability, ergonomics, feasibility, and stability during lithotripsy. In Geavlete et al. [11], Klein et al. [12] and El-Hajj et al. [16], ergonomics was assessed via qualitative feedback.

Author	Year	Ergonomics	System Cost	Learning Curve
Desai et al. [9]	2011	Visual analogue scale - control (8.5), stability (9.0), fragmentation ease (9.2)	NR	NR
Saglam et al. [10]	2014	Significant difference compared to classic FURS, questionnaire score 31.3 vs 5.6	NR	Short introduction (1 hour) to training model
Geavlete et al. [11]	2016	Qualitative assessment - Better comfort for the surgeon	NR	NR
Klein et al. [12]	2021	Qualitative assessment - Reduction in physical stress and musculoskeletal pain	Initial high cost	Proficiency requires >50 procedures
Tokatli et al. [13]	2022	NR	NR	NR
Firori et al. [14]	2023	Surgeon satisfaction questionnaire - 4.6 score, better stability, less fatigue	NR	Learning curve to control the PlayStation-like controller
Laszkiewicz et al. [15]	2024	NR	NR	NR
Salah et al. [1]	2024	NR	NR	NR
El-Hajj et al. [16]	2024	Qualitative assessment - Reduced fatigue, bone and muscle pain	NR	Fast learning curve
Farre et al. [17]	2024	Likert scale questionnaire - 4/5	System cost - 200,000 euros	NR
Landman et al. [18]	2024	NR	NR	NR
Kim et al. [19]	2025	Less fatigue and numbness - Assessed by a scoring system	NR	NR

*Cost efficiency: *There was minimal documentation of the cost of the robot and its effectiveness across the studies. Only Farré et et al. [17] and Klein et al. [12] reported the initial high acquisition and maintenance costs for robotic systems when compared to FURS. The potential long-term cost efficiency can be considered by improving surgeon ergonomics, supporting instrument longevity, and possibly reducing perioperative morbidity.

*Learning curve: *Most studies indicate that the learning curve for robotic flexible ureteroscopy is relatively short, with surgeons achieving proficiency after a limited number of cases. Simulation-based training and intuitive console interfaces facilitate skill acquisition, even for trainees with limited prior experience. However, transitioning between manual to robotic systems may require additional adaptation and operational satisfaction can initially be lower until familiarity is gained, as noted in Klein et al. [12].

Methodological quality and risk of bias assessment

**Figure 5:**
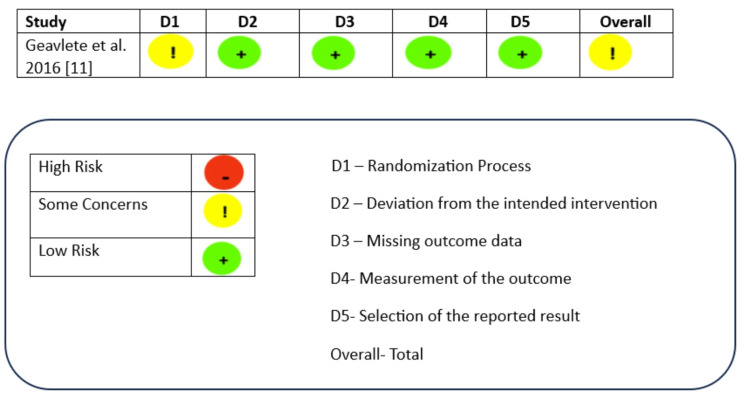
Assessment of risk of bias of the randomised trials using the Cochrane Collaboration Tool - risk of bias (RoB) 2.

Methodological quality was evaluated using the Newcastle-Ottawa Scale (NOS), which employs a star-based scoring system across three domains (Table 5). All studies included in the meta-analysis received three stars in the selection domain, confirming appropriate representation of their respective populations. However, the comparability domain demonstrated limitations due to the absence of control arms in most articles. For the outcome assessment domain, all studies achieved three stars, reflecting rigorous outcome measurement methodologies. The Cochrane collaboration tool for methodological quality review was used to assess the risk of bias for the single randomized controlled trial [11] within the review (Figure 5). Apart from the concern regarding the randomisation of the subjects, the study was of good quality overall in terms of intervention and outcome measurement.

**Table 5:** Newcastle-Ottawa Scale (NOS) risk of bias assessment for included studies

Reference	Selection (max 4)	Comparability (max 2)	Outcome (max 3)	Total Stars (max 9)	Quality Level
Desai et al. (2011) [9]	★★★	-	★★★	6	Moderate
Saglam et al. (2014) [10]	★★★	-	★★★	6	Moderate
Klein et al. (2021) [12]	★★★	-	★★★	6	Moderate
Tokatli et al. (2022) [13]	★★★	-	★★★	6	Moderate
Fiori et al. (2023) [14]	★★★	-	★★★	6	Moderate
Laskiewicz et al. (2024) [15]	★★★	-	★★★	6	Moderate
Salah et al. (2024) [1]	★★★	-	★★★	6	Moderate
El-Hajj et al. (2024) [16]	★★★	-	★★★	6	Moderate
Farré et al. (2024) [17]	★★★	-	★★★	6	Moderate
Landman et al. (2024) [18]	★★★	-	★★★	6	Moderate
Kim et al. (2025) [19]	★★★	-	★★★	6	Moderate

Discussion

RFURS represents a significant advancement in minimally invasive stone management, driven by ongoing innovations in endourological technology [20]. The integration of robotic platforms is intended to address ergonomic limitations and improve procedural accuracy relative to standard FURS [21]. Emerging evidence highlights the potential of RFURS to achieve high SFRs and maintain a favourable safety profile across a broad spectrum of patients. This is the first systematic review and meta-analysis, which provided a comprehensive evaluation of the current literature regarding the efficacy, safety, ergonomics and learning curve of robotic flexible ureteroscopy.

Robotic ureteroscopy has evolved rapidly over the past two decades, building upon the foundational advances in flexible ureteroscope technology since the 1960s [22]. The first clinical application of a robotic flexible ureteroscope was reported in 2011 by Desai et al. [9] using the Sensei-Magellan system, originally designed for cardiac interventions. Although initial models faced technical limitations, subsequent development led to purpose-built platforms specifically for ureteroscopy [23]. Avicenna Roboflex® (ELMED, Ankara, Türkiye) was the world’s first flexible ureteroscopy robot, offering a remote console for precise control of ureteroscope movements, laser and irrigation, with a focus on surgeon ergonomics and radiation safety [10]. Avicenna Roboflex® utilises a master-slave console with integrated laser and basket controls, enabling 220° scope rotation and radiation-free operation. The ILY® system (Sterlab, Vallauris, France) is a wireless, telemanipulated ureteroscope holder compatible with multiple scopes and access sheaths; it enables ±360° rotation, rapid setup (<5 minutes) and allows the surgeon to operate from a seated, radiation-shielded position, significantly reducing fatigue and musculoskeletal strain [23]. One limitation of the ILY®, however, is that using a video-game controller forces the surgeon to learn how to use the control buttons to replicate the expected flexible ureteroscope movement, which might be time-consuming [15]. The Monarch™ platform (Ethicon/Auris Health, USA) is a multispecialty robotic system recently cleared by the Food and Drug Administration (FDA) for urological use, providing a single platform for both ureteroscopic and percutaneous procedures, with robotic arm control for enhanced precision and visualisation [18]. Zamenix R (Roen Surgical Inc., Daejeon, Korea) is specifically designed for retrograde intrarenal surgery, featuring a surgeon console with handle controllers for remote manipulation of the ureteroscope, laser fiber, and stone basket [19]. Zamenix R incorporates gimbal handle controllers for intuitive manipulation, automatic navigation algorithms and safety alarms to prevent oversized stone retrieval. These platforms collectively enhance procedural precision while reducing physical strain through ergonomic design and integrated instrument management.

The pooled SFR of 87.4% (95% CI: 82.7%-92.0%) for RFURS demonstrates comparable or superior efficacy to FURS, with important clinical implications for patient selection and treatment planning. Robotic systems show excellent efficacy with rates approaching 90-95% (Tokatli et al. [13]: 95.5%, El-Hajj et al. [16]: 93.5%). Salah et al. [1] reported 80% vs. 44% SFRs for stones ≤10 mm vs >20 mm, respectively, and no difference was noted in SFR for lower pole calyceal stone. Karagoz et al. reported a SFR of 84.1% for <20 mm stones and 58.33% for >20 mm stones (p=0.008) in classic FURS [24]. The 95.5% SFR in mini-ECIRS [13] suggests robotic systems may excel in challenging anatomical scenarios. Equivalent efficacy supports RFURS as a viable alternative for patients requiring prolonged procedures or complex stone configurations.

Individual study operative times ranged dramatically from 51.0 minutes (Geavlete et al. [11]) to 208.0 minutes (Landman et al. [18]) with a pooled mean of 94.7 minutes. Comparable duration for FURS is recorded (64.5 minutes) [20]. The longer duration can be attributed to the robot docking and setup, which in the study has ranged from 59.6 seconds to 9 minutes. The longer operative time may be due to the learning curve in adopting new technology in the early clinical experience. The influence of different robot platforms has been noticed in our study, demonstrating an average duration of 51-91 minutes in Avicenna Roboflex® and longest in Monarch™ robot where ECIRS was performed for large-volume stones.

The pooled complication rate for RFURS was 10.6% (95% CI: 5.1%-16.1%), indicating a safety profile comparable to that of conventional FURS. These findings highlight key clinical considerations for the integration and optimisation of RFURS in practice. Individual study complication rates varied considerably, from 0.7% (Geavlete et al. [11]) to 33.3% (Farre et al. [17]). Notably, two studies (Geavlete et al. [11] and Tokatli et al. [13]) reported very low complication rates (<0.7%), while others reported higher rates, particularly in smaller studies. The Clinical Research Office of the Endourological Society reported an overall complication rate of 3.5% in 11,885 patients undergoing FURS [25]. A large series by Bas et al. [26] documented 13.3% overall complications (209/1,571 procedures) with 5.9% intraoperative complications, including bleeding (2.5%) and mucosal injury (2.3%). RFURS demonstrates acceptable safety with a 10.6% overall complication rate, though significant inter-study variability warrants further investigation of contributing factors. None of the included studies reported any intraoperative device malfunctions, highlighting the safety and reliability of the device during clinical use.

RFURS is associated with a notably short hospital stay. The median length of stay reported across studies is approximately 9.3 hours (interquartile range (IQR) 5.4-166), with the vast majority of patients (95%) discharged on the same day of surgery [1,14,16]. Only a small minority required overnight admission, and earlier studies reported mean hospital stays of 1.5 to 2.3 days, reflecting increasing adoption of day-care surgery [9]. This short length of stay highlights the minimally invasive nature and rapid recovery profile of RFURS. Retreatment rates following RFURS are low. In one large series (Salah et al. [1]), 73% of patients were stone-free after a single treatment session and tetrafecta (complete clearance, no high-grade complications, no auxiliary procedures, and same-day discharge) was achieved in 70% of cases. Klein et al. [12] reported a retreatment rate of 8.75%, indicating that only a small proportion of patients required additional procedures for residual stones. The better ergonomics lead to precision in the procedure and reduce residual fragments and re-treatment.

RFUS systems offer markedly superior ergonomic performance compared to FURS, addressing critical limitations of traditional FURS. Surgeons report substantially reduced physical strain - particularly in the neck, shoulders, and hands - due to seated console operation, tremor-filtered instrument control and elimination of prolonged static postures. This aligns with Saglam et al. [10], where RFURS reduced surgeon discomfort scores by 40% compared to FURS [10]. These ergonomic advantages not only improve procedural precision but also enhance surgeon sustainability, potentially extending career longevity in high-volume endourology practices. In addition to improved ergonomics, the reduction in radiation exposure is also a highlight for robotic ureteroscopy. The design of the ILY® system facilitates operator positioning behind lead-shielding barriers, thereby enhancing radiation safety without compromising procedural control [27].

The learning curve for RFUS varies significantly by platform but remains manageable overall. Surgeons typically achieve proficiency with the Avicenna Roboflex® after five to 10 cases, with some studies reporting that a short learning curve of as few as five cases is sufficient for experienced endourologists [3]. The ILY® system features an intuitive interface that allows surgeons to become competent in just three to five cases, and clinical studies confirm that urologists familiar with the device controller do not require extensive training [15]. The PlayStation-like controller demands a learning curve, particularly who are not used to gaming [14]. Simulation-based training programs have been shown to reduce skill acquisition time by up to 40%, accelerating the learning process for both novice and experienced users [28]. Early adopters frequently experience lower initial satisfaction, particularly during their first few cases. However, satisfaction improves significantly with increased hands-on experience, as users become more familiar with instrument feedback and system dynamics [29].

Whilst addressing the cost effectiveness of RFURS, only two studies mentioned the high initial cost involved [12,17]. A recent review by Rassweiler-Seyfried et al. [3] highlights that while RFURS offers potential ergonomic and technical advantages, the high initial and ongoing costs of robotic platforms remain a major barrier to widespread adoption. The financial justification for RFURS is currently limited to high-volume specialised centres, and robust cost-effectiveness data comparing with disposable or reusable ureteroscopes are still lacking [3].

Limitations

This meta-analysis is limited by the small number and generally low quality of the included studies, most of which were single-centre and retrospective, increasing the risk of selection and reporting bias. There was considerable heterogeneity across studies in terms of patient selection, surgeon experience, robotic platforms and outcome definitions, which reduces the comparability and generalisability of the results. Additionally, outcome assessment was not standardised, with varying definitions of residual fragment size and different imaging modalities used, potentially affecting the accuracy of reported rates. The analysis also lacked data on complex cases and did not address cost-effectiveness, while the exclusion of non-English and unpublished studies raises the possibility of publication bias. Formal assessment of publication bias (e.g., funnel plot analysis or Egger’s test) was not performed because as with fewer studies, these statistical tools lack sufficient power and may give misleading or inconclusive indications of bias. Therefore, in this context, omitting such an assessment is methodologically appropriate and justified.

Recommendation from authors

We recommend conducting large, multi-centre randomised controlled trials comparing RFURS with conventional ureteroscopy, focusing on effectiveness, safety, ergonomics and cost over a follow-up period longer than 24 months. Outcomes should be standardised using agreed-upon criteria, such as the "tetrafecta" endpoints and consistent definitions of stone-free status based on fragment size and stone location. Further research should also focus on improving technology by adding features like clinically tested haptic feedback, automation for tasks like navigation, stone retrieval, and pressure monitoring to reduce irrigation risks. These steps will help define the true clinical value of RFURS and ensure its practical use in patient care.

## Conclusions

RFURS represents a significant advancement in endourology, offering comparable SFRs (87.4%) and safety profiles (10.6% complications) to conventional FURS while addressing critical ergonomic challenges. Key advantages include reduced surgeon fatigue, improved precision and radiation safety through remote console operation. However, longer operative times and substantial upfront costs remain barriers. The learning curve is platform-dependent with simulation training accelerating the proficiency. Further innovations can collectively promise improved surgeon well-being, procedural efficiency and patient safety, promising robotic ureteroscopy as a key technology for the future of endourology.

## Appendices

Supplementary material

Comprehensive Search Strategy for Systematic Review Databases: Medline (Ovid), Embase (Ovid) and Cochrane Central

Timeframe: January 2010 to June 2025

Filters: Only English; human studies

1. Core Search Terms: PICOS (Population, Intervention, Comparison, Outcome, and Study Design) Components:

  Population: ("Kidney Calculi" OR "Nephrolithiasis" OR "Urolithiasis" OR "Renal Stone*")

  Intervention: ("Robotic Flexible Ureteroscopy" OR "Robot-Assisted Ureteroscopy" OR "RF-URS" OR "Avicenna Roboflex" OR "ILY

                         Robot" OR "Zamenix R")

  Outcomes: Surgical: ("Stone-Free Rate" OR "SFR" OR "Operative Time" OR "Complications" OR "Clavien-Dindo") 

                     Ergonomic: ("Ergonomics" OR "Musculoskeletal Strain" OR "Surgeon Fatigue")

Study Design: ("Randomized Controlled Trial" OR "Cohort Studies" OR "Case Series")

2. Medline (Ovid) Strategy

Kidney Calculi/(Renal Stone* OR Nephrolithiasis OR Urolithiasis) AND Robotic Surgical Procedures/(Robotic Flexible Ureteroscopy OR Robot-Assisted Ureteroscopy OR RFURS) AND (Avicenna Roboflex OR ILY Robot OR Zamenix R) AND (Stone-Free Rate OR SFR OR Operative Time OR Complications) AND (Ergonomics OR Musculoskeletal Strain).

3. Embase (Ovid) Adaptation

Kidney stone/(Renal Stone* OR Nephrolithiasis) AND exp robot-assisted surgery/(Avicenna Roboflex OR ILY OR Zamenix) AND (Stone-Free Rate OR Complication* OR Operative Time) AND (ergonomics/ OR NASA-TLX OR Musculoskeletal Pain)

4. Cochrane Central

 [mh "Kidney Stone"] OR (Renal Stone* OR Nephrolithiasis)  

  AND [mh "Robotics"] OR (Robotic Ureteroscopy OR RF-URS) OR (Avicenna OR ILY OR Zamenix)

  AND (SFR OR "Operative Time" OR Complications)

  AND (Ergonomics OR "Surgeon Fatigue")

5. Supplementary Search Tactics

• Conference Proceedings: European Association of Urology (EAU), American Urological Association (AUA) (2010-2025) using keywords: ("Robotic Ureteroscopy" AND "Kidney Stones")

• Clinical Trial Registries: ClinicalTrials.gov, World Health Organization International Clinical Trials Registry Platform (WHO ICTRP). Search keywords: ("Robotic" AND "Ureteroscopy") + Condition: "Kidney Stones"

• Hand-Searching: References of included studies and relevant reviews.

**Table 6:** Grading of Recommendations Assessment, Development and Evaluation (GRADE) quality assessment results RCT: Randomised control trial.

Outcome	Number of Studies	Study Design	Risk of Bias	Inconsistency	Indirectness	Imprecision	Other Considerations	Overall Quality of Evidence
Stone-Free Rate	12	Prospective, retrospective, single-arm, RCT	Moderate	Low	Low	Moderate	Publication bias possible	Moderate
Complications	10	Prospective, retrospective, single-arm, RCT	Low	Low	Low	Moderate	Consistent reporting	Moderate
Ergonomics	8	Prospective, surveys, single-arm	Moderate	Moderate	High	High	Subjective reporting	Low
Learning Curve	4	Prospective, observational	Moderate	Moderate	High	High	Heterogeneous endpoints	Low
